# Associations of Short-Term Particle and Noise Exposures with Markers of Cardiovascular and Respiratory Health among Highway Maintenance Workers

**DOI:** 10.1289/ehp.1307100

**Published:** 2014-03-19

**Authors:** Reto Meier, Wayne E. Cascio, Andrew J. Ghio, Pascal Wild, Brigitta Danuser, Michael Riediker

**Affiliations:** 1 Institute for Work and Health (Institut universitaire romand de Santé au Travail), University of Lausanne and University of Geneva, Lausanne, Switzerland; 2 Environmental Public Health Division, National Health and Environmental Effects Research Laboratory, U.S. Environmental Protection Agency, Research Triangle Park, North Carolina, USA; 3 Institute for Research and Safety, Vandoeuvre-lès-Nancy, France; 4 Institute of Occupational Medicine, Singapore

## Abstract

Background: Highway maintenance workers are constantly and simultaneously exposed to traffic-related particle and noise emissions, both of which have been linked to increased cardiovascular morbidity and mortality in population-based epidemiology studies.

Objectives: We aimed to investigate short-term health effects related to particle and noise exposure.

Methods: We monitored 18 maintenance workers, during as many as five 24-hr periods from a total of 50 observation days. We measured their exposure to fine particulate matter (diameter ≤ 2.5 μm; PM_2.5_), ultrafine particles, and noise, and the cardiopulmonary health end points: blood pressure, proinflammatory and prothrombotic markers in the blood, lung function, and fractional exhaled nitric oxide (FeNO) measured approximately 15 hr after work. Heart rate variability was assessed during a sleep period approximately 10 hr after work.

Results: PM_2.5_ exposure was significantly associated with C-reactive protein and serum amyloid A, and was negatively associated with tumor necrosis factor α. None of the particle metrics were significantly associated with von Willebrand factor or tissue factor expression. PM_2.5_ and work noise were associated with markers of increased heart rate variability, and with increased high-frequency and low-frequency power. Systolic and diastolic blood pressure on the following morning were significantly associated with noise exposure after work, and nonsignificantly associated with PM_2.5_. We observed no significant associations between any of the exposures and lung function or FeNO.

Conclusions: Our findings suggest that exposure to particles and noise during highway maintenance work might pose a cardiovascular health risk. Actions to reduce these exposures could lead to better health for this population of workers.

Citation: Meier R, Cascio WE, Ghio AJ, Wild P, Danuser B, Riediker M. 2014. Associations of short-term particle and noise exposures with markers of cardiovascular and respiratory health among highway maintenance workers. Environ Health Perspect 122:726–732; http://dx.doi.org/10.1289/ehp.1307100

## Introduction

Long-term exposures to particulate matter (PM) and noise have both been associated with cardiovascular diseases such as ischemic heart disease and hypertension (Babisch 2011; Brook et al. 2010). Although particle and noise exposure frequently occur concomitantly, only a few recent epidemiologic studies have controlled for both factors (Beelen et al. 2009; de Kluizenaar et al. 2007; Fuks et al. 2011; Huss et al. 2010).

PM-related health effects have been widely studied, and exposure to PM has been associated with cardiopulmonary diseases, which increase hospitalization and premature deaths throughout the world (Brook et al. 2010; Schwartz 1999; Schwartz et al. 2002). Ultrafine particles (UFP), with diameters < 100 nm, are considered to play an important role in triggering particle-related health effects because of their small size and large surface area. There is evidence of effects of noise on the cardiovascular system: Noise exposure in both residential and occupational settings has been associated with hypertension (Brook 2007; de Kluizenaar et al. 2007; Fuks et al. 2011; van Kempen and Babisch 2012; van Kempen et al. 2002), ischemic heart disease, and myocardial infarction (Davies et al. 2005; Huss et al. 2010; Selander et al. 2009).

Road traffic is an important source of PM and noise emissions, both of which have been associated with cardiovascular effects. Alterations in heart rate variability (HRV) and vascular inflammation (Riediker et al. 2004; Wu et al. 2010), as well as progression of atherosclerosis (Bauer et al. 2010; Künzli et al. 2010) have been attributed to traffic-related PM and at levels lower than the current annual PM_2.5_ (PM with diameter ≤ 2.5 μm) air quality standard in the United States (Adar et al. 2013). Myocardial infarction (Babisch et al. 2005; Bigert et al. 2003; Peters et al. 2004) and elevated cardiovascular morbidity and mortality in the general population (Babisch 2006, 2008; Hoek et al. 2002; Janssen et al. 2002) have been associated with traffic in general, as well as with PM and/or noise emissions from traffic. Because of the simultaneous exposure it is often difficult to disentangle particle- and noise-related health effects.

We recently described how highway maintenance workers are frequently exposed to air pollutants and noise originating from road traffic and work equipment (Meier et al. 2013a). Their mixed exposure to particles and noise may contribute to an increased susceptibility to the development or exacerbation of heart and vascular diseases. To investigate short-term health effects related to particles and noise, we estimated associations of exposures to PM_2.5_, UFP, and noise with various cardiopulmonary health end points. We hypothesized that particle exposure would lead to increased levels of proinflammatory and prothrombotic markers in the blood. We also expected particle- and noise-related changes in heart rate variability and an association of blood pressure elevation and noise. Because cardiovascular effects for both exposure types have been described previously, we were interested in investigating mutually adjusted health effects.

## Methods

*Study design*. To investigate short-term health effects of exposure to PM and noise, we carried out repeated measurements on 18 highway maintenance workers. Exposure and health assessments were conducted on 50 days between May 2010 and February 2012 in collaboration with the Swiss Road Maintenance Services on highways in western Switzerland. For work shifts of 38 days, two co-located subjects were equipped with personal measurement equipment, whereas only one subject was equipped for work shifts of 12 days. Healthy, nonsmoking male maintenance workers from 10 maintenance centers were offered participation in the study. Health criteria for exclusion were high blood pressure (systolic/diastolic > 140/90 mmHg), cardiopulmonary health problems, acute allergies, diabetes, and obesity [body mass index (BMI) > 32]. Exposure to PM_2.5_, UFP, noise, and gaseous co-pollutants was assessed during five nonconsecutive work shifts. To control for post-work-shift exposure, personal PM_2.5Realtime_ and noise exposure measurement was continued after end of work (around 1700 hours) until the next morning. PM_2.5Realtime_ measurements after work have been used only in a sensitivity analysis and not for the reported associations.

Exposure parameters were compared with HRV assessed during a sleep period approximately 10 hr after work and with further health end points that were assessed on the following morning, approximately 15 hr after work. The study was approved by the Ethics Committee from the University of Lausanne, and all research volunteers provided written informed consent.

*Exposure assessment*. The exposure assessment was presented in detail elsewhere (Meier et al. 2013a). Briefly, personal real-time PM_2.5_ measurements (PM_2.5Realtime_; 1-min resolution) were made using a personal DataRam pDR1000 real-time particulate monitor (Thermo Scientific, Waltham, MA, USA) attached on the subject’s back. PM_2.5_ was also measured gravimetrically (PM_2.5Mass_) at the work site using PTFE filters (#225-1709; SKC Inc., Eighty Four, PA, USA) and a Leland Legacy sampling pump (SKC Inc.) with a flow rate of 10 L/min. UFP number concentrations and the lung-deposited surface area (LDSA; particle surface area concentration deposited in the lung) were measured at the work site using a miniDiSC (Fierz et al. 2011), a method that has been shown to provide reliable results under highway conditions in the 16–300 nm size range (Meier et al. 2013b). We chose LDSA as the UFP exposure metric for reporting associations with health outcomes because surface area is an important determinant for particle reactivity (Duffin et al. 2002). Gaseous co-pollutants were measured at the work site: carbon monoxide (CO) with the T15n CO monitor (Langan Products, San Francisco, CA, USA); nitrogen dioxide (NO_2_) and ozone (O_3_) concentrations with diffusive samplers from Passam AG (Männedorf, Switzerland). O_3_ concentrations did not reach the detection limit (L_O3_) of 7.6 ppb for an 8-hr measurement during 22 of a total of 50 work shifts. For models including O_3_ (only in sensitivity analyses) values below the detection limit were replaced with L_O3_/2 (3.8 ppb). Temperature and humidity were measured with HOBO data loggers (U12-012; Onset Computer Corporation, Cape Cod, MA, USA). Noise was measured with personal noise dosimeters (Type 4500; Bruel & Kjaer, Nærum, Denmark) attached to the subjects. Measurements were made in the range from 70 to 140 dB(A) during work shifts and from 50 to 100 dB(A) for the continued postwork assessment until the next morning. Noise levels were corrected for periods when hearing protection was used: a 25-dB correction during use of acoustic earmuffs [SNR (single number rating) 30] and a 20-dB correction for preformed earplugs (SNR 25).

A detailed description about handling of missing real-time exposure data has been provided previously (Meier et al. 2013a). Briefly, missing real-time particle data were replaced by estimates based on correlated particle measurements extrapolated to the distribution of the missing pollutant for the same subject, activity, and type of work site: For PM_2.5Realtime_, 0.5% missing, estimates based on parallel PM_2.5Realtime_ measurement, and, if not available, on UFP particle number concentration (PNC). For UFP, 4.8% missing, estimates based on PM_2.5Realtime_. Estimations for missing noise data (3.6%) were based on the parallel noise measurement and, if those were not available, on data from the same subject, activity, and type of work site. Missing noise data for six home-based measurements and one work-shift measurement (microphone or battery failures) could not be replaced with estimations and were not considered for analysis. Two PM_2.5Realtime_ measurements stopped early (battery failure) and did not include full duration of post-work assessment.

*Health assessment*. Before starting maintenance work and exposure assessment in the morning, the subjects were equipped with Holter H12+ Digital Recorders (Mortara Instrument, Inc., Milwaukee, WI, USA) for continuous recording of their electrocardiogram (ECG). Blood pressure, fractional exhaled nitric oxide (FeNO), and lung function were measured during a health assessment on the following morning—approximately 15 hr after work. Also, a blood sample was taken by a trained nurse for subsequent assessment of blood markers, and subjects provided information about their health status and drug intake.

Fresh blood serum and plasma samples were centrifuged and stored in a cold box at approximately 5°C until they were frozen at –80°C after a maximum of 2 hr. Frozen samples were shipped to and analyzed at the National Health and Environmental Effects Research Laboratory at the U.S. Environmental Protection Agency (Chapel Hill, NC, USA). Blood markers in serum were quantified with electrochemiluminescence detection: serum levels of interleukin 6 (IL-6) and tumor necrosis factor α (TNFα) were assessed with the Human ProInflammatory-4 II Ultra-Sensitive Kit (Meso Scale Discovery, Rockville, MD, USA). C-reactive protein (CRP) and serum amyloid A (SAA) in serum were measured using the Human Vascular Injury II Kit (Meso Scale Discovery). von Willebrand factor (vWF) was quantified in plasma with the Asserachrom® VWF:Ag ELISA kit (Diagnostica Stago, Inc., Parsippany, NJ, USA), and tissue factor (plasma) was measured with the Human Coagulation Factor III/Tissue Factor Quantikine ELISA (R&D Systems, Inc., Minneapolis, MN, USA).

ECG data were processed with H-Scribe+ software (Mortara Instrument, Inc.) and inspected manually by an experienced cardiologist (W.E.C.). Data were subsequently processed with Super ECG Software provided by David Mortara (Mortara Instrument, Inc.). Particle and noise related effects on HRV were estimated for the 5-min period between 0200 and 0400 hours when subjects were asleep and had the lowest mean heart rate. This nightly time window was chosen because it reflects a well-defined resting period when subjects were in horizontal position. The following HRV outcomes were used: standard deviation of NN intervals (SDNN), ratio of the number of pairs of adjacent NN intervals differing by > 50 msec to the total number of NN intervals (pNN50), root mean square of the differences of successive NN intervals (RMSSD), low-frequency power (LF; 0.04–0.15 Hz), and high-frequency power (HF; 0.14–0.40 Hz). Blood pressure was measured the following morning with the automatic blood pressure monitor M10-IT (Omron Healthcare Europe, Hoofddorp, the Netherlands). The average of three successive measurements within 5 min has been used. Lung function was measured with the EasyOne Worldspirometer (NDD Medizintechnik, Zurich, Switzerland) in the “FVC [forced vital capacity] expiratory” test mode; test procedure was according to American Thoracic Society standards for forced expiratory volume in 1 sec (FEV_1_) and FVC test procedure (Miller et al. 2005). Expiratory air for FeNO analysis was collected with an offline collection kit (ECO MEDICS AG, Duernten, Switzerland) (Schiller et al. 2009). Samples were taken in triplicate and analyzed within 6 hr of sampling. Analysis was performed with the EcoMedics CDL-88-Analyzer.

*Statistical analysis*. Health end points were compared with particle and noise exposure averaged over the preceding work shift and noise exposure during the time period after work. Linear mixed-effects regression models with subject-specific random intercepts were used to estimate exposure-related health effects clustered in individuals. We estimated mutually adjusted associations of particles and noise exposures by including both parameters in the same models. Separate models were used for particle exposure metrics: PM_2.5Realtime_ and PM_2.5Mass_ (10-μg/m^3^ increase), UFP PNC (10,000-particles/cm^3^ increase), UFP LDSA (10-μm^2^/cm^3^ increase); each model also included separate continuous variables for noise at work and noise after work, with associations estimated for a 1-dB(A) increase in each noise exposure metric. All models were adjusted for age and BMI as continuous variables. Confounding of other untransformed continuous covariates (temperature, humidity, NO_2_, O_3_, CO) was assessed by sensitivity analyses in which models were adjusted for these variables. All HRV outcomes other than pNN50 and the IL-6, CRP, and SAA blood markers were normalized by natural log-transformation. We used a *p*-value < 0.05 to define statistical significance. All statistical models were calculated using STATA release 12 (StataCorp LP, College Station, TX, USA).

## Results

For the analysis of short-term health effects of particle and noise exposure we used data from repeated measurements on 18 healthy non-smoking male highway maintenance workers. Subjects participated in five repeated measurements (one subject only in three repetitions). Subjects were between 31 and 59 years old (mean, 46 years). Their weights ranged from 78 to 107 kg (mean, 82.4 kg) and their heights from 165 to 187 cm (mean, 175 cm), with BMIs between 21.8 and 31.1 kg/m^2^ (mean, 26.7 kg/m^2^). Two of the subjects were being treated with ACE (angiotensin-converting-enzyme) inhibitor for high blood pressure; one subject took a low-dose aspirin daily. A sensitivity analysis excluding these subjects did not change associations with the investigated health end points. Three measurements were excluded post hoc because subjects did not meet the inclusion criteria of being healthy (reported cold symptoms, cold medication, CRP levels > 15 mg/L). One particular work shift during which two measurements were conducted was excluded because particle exposure was very high (PM_2.5Realtime_ > 500 μg/m^3^; PM_2.5Mass_ > 300 μg/m^3^; LDSA > 600 μm^2^/cm^3^) and did not represent a standard exposure (> 4 × SD higher than the mean). The following health data were missing: HRV data of five measurements (memory card or battery failure; one with bad ECG signal), blood data of six measurements (subjects refused blood withdrawal), and a plasma sample for vWF and tissue factor analysis of one measurement (lost during analysis). A total of 77 observations for which particle and noise exposure as well as health parameters were available were used for blood pressure models, 73 observations for HRV models, and 71 observations for blood marker models (70 for vWF and tissue factor).

Summary statistics with the exposure averages used to assess associations with health outcomes are shown in Table 1. As a consequence of the varied work activities, we observed a high variability in particle and noise exposure. Particle concentrations and noise levels were highest for work shifts that included the use of specific working equipment (hand-held string trimmers, chain saws, pneumatic hammers). PM_2.5_ levels after work were considerably lower than during work, and their contribution to the total particle dose was minor. Noise measurements after work were characterized by noise levels in the early evening; nighttime noise rarely reached the lower measurement limit of 50 dB(A). Spearman correlations of particles and noise during work were low (ρ ~ 0.3 for PM_2.5_ and noise) to moderate (ρ = 0.5 for PNC and noise). LDSA, a measure of exposure to UFP, was highly correlated to the UFP particle number concentration (PNC) (Pearson correlation *r* = 0.96). A more detailed description of exposure to air pollutants and noise during highway maintenance work has been described previously (Meier et al. 2013a). The slight differences between the means reported in Table 1 and the means reported in our previous publication are attributable to excluded observations because of missing or invalid health data.

**Table 1:** Summary statistics of exposure parameters used to assess associations between exposure and health outcomes.

Exposure	Mean ± SD	Minimum	Maximum	No. of observations^*a*^
PM_2.5Realtime_ work (μg/m^3^)	65.7 ± 69.9	7.3	347.8	77
PM_2.5Realtime_ after work (μg/m^3^)^*b*^	22.9 ± 19.5	0.2	81.3	75
PM_2.5Mass_ work (μg/m^3^)	56.1 ± 39.0	20.3	186.9	48
UFP PNC work (particles/cm^3^)	75,699 ± 81,761	15,524	331,683	48
UFP LDSA work (μm^2^/cm^3^)^*c*^	111.6 ± 86.8	31.5	385.5	48
*L*_eq_ work (dB[A])^*d*^	81.0 ± 3.6	73.3	91.6	77
*L*_eq_ after work (dB[A])	65.8 ± 5.8	56.4	85.0	77
*L*_eq_, equivalent continuous noise level. ^***a***^Number of total personal exposure assessments (PM_2.5Realtime_ and *L*_eq_) and total exposure assessment at work site (PM_2.5Mass_, UFP PNC, and UFP LDSA). Exposure measured at work site was assigned to two subjects when two co-located subjects were measured during the same work shift. Due to missing health data, not all observations were used in all models: a total of 77 observations were used for blood pressure models, 73 observations for HRV models, and 71 observations for blood marker models. ^***b***^PM_2.5Realtime_ measurements after work have only been used in sensitivity analyses and not for the reported associations. ^***c***^Lung-deposited surface area of ultrafine particles. ^***d***^*L*_eq_ over full work shift corrected for the use of ear protection.				

All health parameters were within a normal range; summary statistics of health end points are provided in Table 2. Coefficients of mixed-effects regression models used to estimate associations between particles and blood markers (with adjustment for work and after work noise) are shown in Table 3. PM_2.5Mass_ was significantly and positively associated with CRP and SAA concentrations [percent increases associated with a 10-μg/m^3^ increase in exposure of 5.56% (95% CI: 1.05, 10.27%) and 3.56% (95% CI: 0.04, 7.21%), respectively] and negatively associated with TNFα (–0.60%; 95% CI: –1.15, –0.04%). None of the proinflammatory or prothrombotic markers were significantly associated with UFP LDSA (Table 3) or PNC (data not shown). Work noise was not significantly associated with any of the blood markers (*p* > 0.3), and associations with particle exposures did not change when models were not adjusted for work or after work noise (data not shown). Noise after work was significantly associated with vWF (increase of 1.48%; 95% CI: 0.40, 2.56% per 1 dB; model adjusted for PM_2.5Realtime_ and work noise) but not any of the other blood markers (data not shown).

**Table 2:** Summary statistics of health end points used to assess associations between exposure and health outcomes.

Outcome	Mean ± SD	Minimum	Maximum	No. of observations
Blood pressure (mmHg)
Systolic	122.2 ± 13.4	95.0	154.0	77
Diastolic	78.1 ± 8.6	62.0	102.0	77
Proinflammatory and prothrombotic blood markers
Serum IL-6 (ng/L)	0.54 ± 0.29	0.17	1.46	71
Serum TNFα (ng/L)	3.79 ± 1.00	1.16	6.52	71
Serum CRP (mg/L)	2.09 ± 1.64	0.19	7.24	71
Serum SAA (mg/L)	4.63 ± 4.01	0.85	17.53	71
Plasma vWF (%)	109.22 ± 39.57	37.40	207.00	70
Plasma tissue factor (ng/L)	74.84 ± 29.77	30.90	177.90	70
HRV parameters
Mean HR (beats/min)	54.4 ± 7.7	42.0	79.0	73
SDNN (msec)	82.7 ± 46.9	25.0	226.0	73
pNN50 (%)	28.7 ± 24.8	0.0	87.0	73
RMSSD (msec)	73.8 ± 66.1	8.0	279.0	73
High frequency power (msec^2^)	3,325 ± 6,261	23	32,544	73
Low frequency power (msec^2^)	3,041 ± 2,921	220	14,790	73
Ratio HF/LF	2.9 ± 2.5	0.2	11.3	73
Lung parameters
FeNO (ppb)	18.6 ± 6.3	7.4	38.7	77
FVC (L)	4.8 ± 0.5	3.7	6.1	77
FEV_1_ (L)	3.8 ± 0.4	2.8	4.7	77
Blood pressure, blood markers, and lung parameters were assessed in the morning after the day of exposure assessment. HRV was assessed during a sleep period approximately 10 hr after work.				

**Table 3:** Associations of particle exposures during work and proinflammatory and prothrombotic markers in the blood [percent differences (95% CI)].^*a*^

Outcome	PM_2.5Realtime_	PM_2.5Mass_	LDSA
IL-6	–1.18 (–2.60, 0.26)	–1.52 (–3.98, 1.00)	–0.65 (–1.98, 0.70)
TNFα	–0.25 (–0.58, 0.08)	–0.60 (–1.15, –0.04)	0.02 (–0.31, 0.35)
CRP	1.97 (–0.62, 4.62)	5.56 (1.05, 10.27)	1.38 (–0.88, 3.70)
SAA	1.23 (–0.79, 3.29)	3.56 (0.04, 7.21)	1.00 (–0.88, 2.91)
vWF	0.30 (–0.55, 1.15)	0.41 (–1.06, 1.88)	0.17 (–0.66, 0.99)
Tissue factor	–0.96 (–2.24, 0.32)	–0.56 (–2.80, 1.69)	–0.84 (–2.05, 0.37)
LDSA, lung-deposited surface area of UFP. ^***a***^Estimates from linear mixed-effects regression models with subject-specific random intercepts to account for repeated observations. All models were adjusted for noise exposure at work, noise exposure after work, age, and BMI. Point estimates represent estimated percent changes in natural log (ln)–transformed outcomes with a 10-μg/m^3^ increase in PM_2.5Realtime_ and PM_2.5Mass_, and a 10-μm^2^/cm^3^ increase in LDSA. Percent changes of TNFα, vWF, and tissue factor, which have not been ln-transformed, were calculated in reference to the mean.			

**Figure 1:**
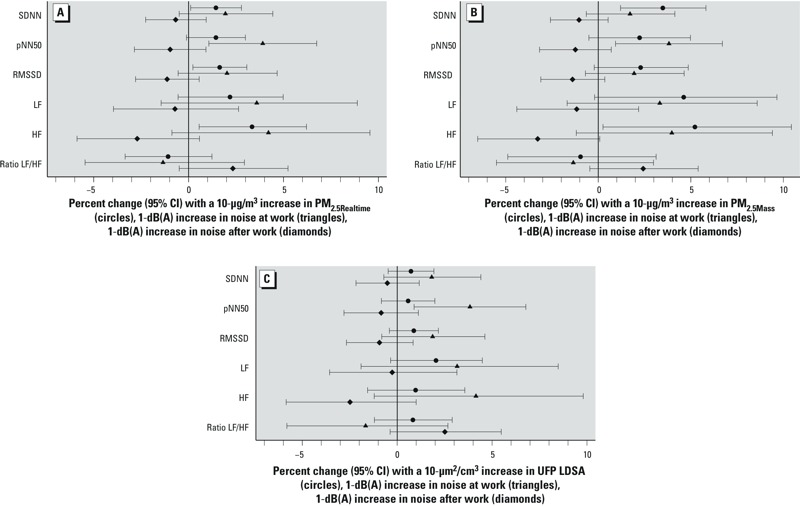
Mutually adjusted associations of particle exposures for PM_2.5Realtime_ (*A*), PM_2.5Mass _(*B*), and UFP LDSA (*C*), noise exposure during work, and noise exposure after work with HRV (measured during a sleep period approximately 10 hr after work). Estimates were from linear mixed-effects regression models with subject-specific random intercepts to account for repeated observations. All models have been adjusted for age and BMI. Percent change of pNN50, which has not been ln-transformed, was calculated in reference to the mean.

In general, 10-μg/m^3^ increases in both PM_2.5_ exposure metrics, and a 1 dB(A) increase in noise at work, were associated with increased HRV, as indicated by positive associations with SDNN, pNN50, and RMSSD (Figure 1). In contrast, a 1-dB(A) increase in noise after work was associated with nonsignificant decreases in HRV. PM_2.5_ exposures and noise at work were significantly associated with HF and also positively associated with LF, with no association with the LF/HF ratio. Noise after work was associated with nonsignificant decreases in HF and increases in the LF/HF ratio. Patterns of associations were similar for UFP LDSA, though point estimates were closer to the null. Associations were comparable for particle exposures based on models that were not adjusted for noise, and for noise exposures based on models that were not adjusted for particles (data not shown). Associations with UFP PNC (data not shown) were similar to those for UFP LDSA.

**Figure 2:**
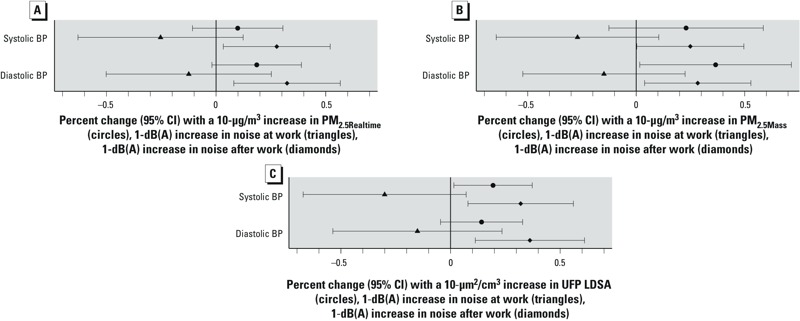
Mutually adjusted associations of particle exposures for PM_2.5Realtime_ (*A*), PM_2.5Mass _(*B*), and UFP LDSA (*C*), noise exposure during work, and noise exposure after work with systolic and diastolic blood pressure measured in the morning approximately 15 hr after work. Estimates were from linear mixed-effects regression models with subject-specific random intercepts to account for repeated observations. All models have been adjusted for age and BMI. Percent changes were calculated in reference to the mean blood pressure.

PM_2.5_, UFP LDSA, and noise after work were positively associated with systolic and diastolic blood pressure the next morning, whereas work noise showed nonsignificant negative associations with blood pressure (Figure 2). Effect estimates were similar for particles without adjustment for noise, and for noise without adjustment for particles (data not shown). However, LDSA was no longer significantly associated with systolic blood pressure when modeled without adjustment for noise (data not shown).

None of the particle exposure metrics were significantly associated with lung function measures (FEV_1_ and FVC) or FeNO (data not shown). We did not estimate associations between noise and lung function.

In a series of sensitivity analyses, we observed that adjusting our models for temperature, humidity, NO_2_, O_3_, or CO did not result in a change in estimated associations between the health outcomes and particles or noise. Associations between co-pollutants and health outcomes were not significant (data not shown). However, associations with all particle metrics were close to the null for all inflammation markers and HRV parameters when two outlier observations with very high particle levels (PM_2.5Realtime_ > 500 μg/m^3^; PM_2.5Mass_ > 300 μg/m^3^; LDSA > 600 μm^2^/cm^3^) were included in the models (data not shown). The origin of these high particle levels is uncertain, but may be related to hot and dry conditions causing elevated dispersion of soil dust during mowing. Furthermore we could see that considering personal PM_2.5Realtime_ averaged over work shift and time at home, instead of considering work shift PM_2.5Realtime_ levels, did not change the findings for PM_2.5Realtime_ (data not shown). Omitting correction of work noise for the use of ear protection resulted in smaller, less significant effect estimates of work noise on HRV (no changes in effect estimates for particles and noise after work) (data not shown). Estimates for blood pressure with/without ear protection were far from significance in either case.

## Discussion

In this study we investigated associations of short-term particle and noise exposures during highway maintenance work with markers of cardiovascular and pulmonary health. Repeated measurements—including the assessment of the particle and noise exposure, as well as selected health end points—were conducted on a sample of 18 healthy, nonsmoking male maintenance workers. PM_2.5_ exposures were positively associated with acute-phase inflammation markers in the blood, and particle and noise exposures at work were associated with higher HRV. Elevated noise exposure during recreational time after work was associated with higher systolic and diastolic blood pressure. We did not observe any evidence of short-term effects of particle exposures on lung function or nitric oxide concentrations in exhaled air.

The positive association of PM_2.5_ with CRP and SAA, two inflammation markers that have been related to arteriosclerosis and other cardiovascular diseases (Johnson et al. 2004; Ridker et al. 2000), is consistent with previous reports of associations between air pollution and traffic exposure with acute-phase inflammation (Peters et al. 2001; Riediker et al. 2004; Rioux et al. 2010). Contrary to our expectations, PM_2.5_ exposure was negatively associated with TNFα and IL-6 concentrations (statistically significant for TNFα). This may be a matter of timing. It has been shown that the TNFα and IL-6 response in rats exposed to diesel did not occur until 24–48 hr after exposure (Kooter et al. 2010). However, the observed negative associations may have been caused by chance or systematic error.

Particle exposures and work noise were both associated with higher HRV during the recovery period in the night. The nightly time window was chosen because it reflects a well-defined resting period when subjects were in horizontal position. Interestingly, particle and work noise were associated with higher HRV independent of each other, based on mutually adjusted models. The increase in high-frequency power and RMSSD suggests changes in vagal activity, which is a major contributor of the HF component (Malik 1996). However, we have seen positive associations with HF and LF power resulting in null associations with the LF/HF ratio. Hence, a reciprocal relationship between sympathetic and parasympathetic balance does not appear to be present under the present conditions similar to the positive associations between ultrafine concentrated ambient particles and HF and LF power reported by a recent study (Samet et al. 2009). Others reported increased vagal activity after exposure to fine particles, characterized by increased RMSSD and decreased SDANN (Pope et al. 1999) and increased HF variability and decreased LF/HF ratio (Riediker et al. 2004). However, particle exposures have often been associated with lower HRV (Gold et al. 2000; Gong et al. 2004; He et al. 2011; Huang et al. 2013; Liao et al. 1999; Magari et al. 2001; Pieters et al. 2012; Weichenthal et al. 2011), and some studies reported evidence of variable effects depending on subjects or particle sizes (Devlin et al. 2003; Timonen et al. 2006; Yeatts et al. 2007). Associations of particle exposures and HRV may be influenced by age, cardiovascular health history, or genetic background, as well as the duration of exposure, timing of the clinical evaluation, and the composition and size of particles. However, there is no clear pattern across these studies that can explain the contradictory results. Timing may have played an important role in regard to the associations between work noise, noise after work, and HRV. A recent study describing immediate changes in HRV changes after noise exposure showed that SDNN was positively associated with concurrent noise > 65 dB(A), but negatively to noise exposure lagged by 5–15 min (Kraus et al. 2013).

Noise exposure after work was significantly associated with higher blood pressure measured on the following morning. Work noise was associated with lower blood pressure, though associations were imprecise and not statistically significant. As for HRV, it is important to consider the time lag of these outcomes. The time point of evaluation may not reflect acute effects of noise exposure of the day before. A few recent studies have reported associations between occupational noise exposure and blood pressure and cardiovascular diseases, although their results are contradictory (Gan et al. 2011; Tomei et al. 2010; van Kempen et al. 2002). However, associations of noise and cardiovascular health outcomes were more commonly linked to traffic and aircraft noise at home and during recreational periods outside occupational settings (Babisch 2011; Dratva et al. 2012; Eriksson et al. 2007; Jarup et al. 2008). The type of noise sources affects noise perception and seems to be an important determinant for noise specific health effects (Babisch 2011). Work noise was primarily dominated by working equipment and secondly by road traffic. We do not have information about the type of noise sources for the period after work. After-work noise was characterized by events in the early evening and might have been strongly influenced by noise caused by the subjects themselves (e.g., hobby, music, televison). However, we cannot differentiate it from environmental noise, and we cannot exclude that disturbing night noise below the lower measurement limit of 50 dB(A) confounded associations with health outcomes. Changes in effect estimates by omitting correction of work noise for ear protection show that controlling for this is important.

The similar effect estimates of particle exposure models adjusted for noise and of noise exposure models adjusted for particles are in line with recent publications that did not see changes when controlling for both factors. Noise did not change effect estimates for an elevated risk for high blood pressure and cardiovascular mortality attributed to PM_2.5_, black smoke, and traffic intensity (Beelen et al. 2009; Fuks et al. 2011). Associations between aircraft noise and death from myocardial infarction were not attenuated by adjusting for PM_10_ (PM with diameter ≤ 10 μm) (Huss et al. 2010). Risk estimates for hypertension in relation to traffic noise did not change significantly when adjusted for PM_10_ (de Kluizenaar et al. 2007). However, a recent study investigating combined effects on HRV reported stronger associations between traffic related air pollution and HRV when noise levels were > 65.6 dB(A) (Huang et al. 2013).

## Conclusions

In this study we observed higher acute-phase inflammation markers CRP and SAA as well as a decrease in TNFα in association with PM_2.5_. PM_2.5_ and work noise were independently associated with higher HRV during the night after a work shift. Noise levels during recreational time after work were positively associated with blood pressure measurements taken the following morning. Our findings suggest that exposure to particles and noise at the workplace might pose a cardiovascular health risk, as evidenced by associated increases in proinflammatory biomarkers. It is therefore important to make efforts to reduce these exposures.

## References

[r1] Adar SD Sheppard L Vedal S Polak JF Sampson PD Diez Roux AV 2013 Fine particulate air pollution and the progression of carotid intima-medial thickness: a prospective cohort study from the multi-ethnic study of atherosclerosis and air pollution. PLoS Med 10 e1001430; 10.1371/journal.pmed.1001430 23637576 PMC3637008

[r2] Babisch W (2006). Transportation noise and cardiovascular risk: updated review and synthesis of epidemiological studies indicate that the evidence has increased.. Noise Health.

[r3] Babisch W (2008). Road traffic noise and cardiovascular risk.. Noise Health.

[r4] Babisch W (2011). Cardiovascular effects of noise.. Noise Health.

[r5] Babisch W, Beule B, Schust M, Kersten N, Ising H (2005). Traffic noise and risk of myocardial infarction.. Epidemiology.

[r6] Bauer M, Moebus S, Mohlenkamp S, Dragano N, Nonnemacher M, Fuchsluger M (2010). Urban particulate matter air pollution is associated with subclinical atherosclerosis: results from the HNR (Heinz Nixdorf Recall) study.. J Am Coll Cardiol.

[r7] Beelen R, Hoek G, Houthuijs D, van den Brandt PA, Goldbohm RA, Fischer P (2009). The joint association of air pollution and noise from road traffic with cardiovascular mortality in a cohort study.. Occup Environ Med.

[r8] Bigert C, Gustavsson P, Hallqvist J, Hogstedt C, Lewne M, Plato N (2003). Myocardial infarction among professional drivers.. Epidemiology.

[r9] Brook RD (2007). Why physicians who treat hypertension should know more about air pollution.. J Clin Hypertens (Greenwich).

[r10] Brook RD, Rajagopalan S, Pope CA, Brook JR, Bhatnagar A, Diez-Roux AV (2010). Particulate matter air pollution and cardiovascular disease: an update to the scientific statement from the American Heart Association.. Circulation.

[r11] Davies HW, Teschke K, Kennedy SM, Hodgson MR, Hertzman C, Demers PA (2005). Occupational exposure to noise and mortality from acute myocardial infarction.. Epidemiology.

[r12] de Kluizenaar Y, Gansevoort RT, Miedema HM, de Jong PE (2007). Hypertension and road traffic noise exposure.. J Occup Environ Med.

[r13] Devlin RB, Ghio AJ, Kehrl H, Sanders G, Cascio W (2003). Elderly humans exposed to concentrated air pollution particles have decreased heart rate variability.. Eur Respir J Suppl.

[r14] Dratva J Phuleria HC Foraster M Gaspoz JM Keidel D Künzli N 2012 Transportation noise and blood pressure in a population-based sample of adults. Environ Health Perspect 120 50 55; 10.1289/ehp.1103448 21885382 PMC3261938

[r15] Duffin R, Tran CL, Clouter A, Brown DM, MacNee W, Stone V (2002). The importance of surface area and specific reactivity in the acute pulmonary inflammatory response to particles.. Ann Occup Hyg.

[r16] Eriksson C, Rosenlund M, Pershagen G, Hilding A, Ostenson CG, Bluhm G (2007). Aircraft noise and incidence of hypertension.. Epidemiology.

[r17] Fierz M, Houle C, Steigmeier P, Burtscher H (2011). Design, calibration, and field performance of a miniature diffusion size classifier.. Aerosol Sci Technol.

[r18] Fuks K Moebus S Hertel S Viehmann A Nonnemacher M Dragano N 2011 Long-term urban particulate air pollution, traffic noise, and arterial blood pressure. Environ Health Perspect 119 1706 1711; 10.1289/ehp.1103564 21827977 PMC3261981

[r19] Gan WQ, Davies HW, Demers PA (2011). Exposure to occupational noise and cardiovascular disease in the United States: the National Health And Nutrition Examination Survey 1999–2004.. Occup Environ Med.

[r20] Gold DR, Litonjua A, Schwartz J, Lovett E, Larson A, Nearing B (2000). Ambient pollution and heart rate variability.. Circulation.

[r21] Gong H, Linn WS, Terrell SL, Clark KW, Geller MD, Anderson KR (2004). Altered heart-rate variability in asthmatic and healthy volunteers exposed to concentrated ambient coarse particles.. Inhal Toxicol.

[r22] He F, Shaffer ML, Li X, Rodriguez-Colon S, Wolbrette DL, Williams R (2011). Individual-level PM_2.5_ exposure and the time course of impaired heart rate variability: the APACR study.. J Expo Sci Environ Epidemiol.

[r23] Hoek G, Brunekreef B, Goldbohm S, Fischer P, van den Brandt PA (2002). Association between mortality and indicators of traffic-related air pollution in the Netherlands: a cohort study.. Lancet.

[r24] Huang J, Deng F, Wu S, Lu H, Hao Y, Guo X (2013). The impacts of short-term exposure to noise and traffic-related air pollution on heart rate variability in young healthy adults.. J Expo Sci Environ Epidemiol.

[r25] Huss A, Spoerri A, Egger M, Röösli M, Swiss National Cohort Study Group (2010). Aircraft noise, air pollution, and mortality from myocardial infarction.. Epidemiology.

[r26] Janssen NAH, Schwartz J, Zanobetti A, Suh HH (2002). Air conditioning and source-specific particles as modifiers of the effect of PM_10_ on hospital admissions for heart and lung disease.. Environ Health Perspect.

[r27] Jarup L Babisch W Houthuijs D Pershagen G Katsouyanni K Cadum E 2008 Hypertension and exposure to noise near airports: The HYENA study. Environ Health Perspect 116 329 333; 10.1289/ehp.10775 18335099 PMC2265027

[r28] Johnson BD, Kip KE, Marroquin OC, Ridker PM, Kelsey SF, Shaw LJ (2004). Serum amyloid A as a predictor of coronary artery disease and cardiovascular outcome in women: the National Heart, Lung, and Blood Institute-Sponsored Women’s Ischemia Syndrome Evaluation (WISE).. Circulation.

[r29] Kooter IM Gerlofs-Nijland ME Boere AJ Leseman DL Fokkens PH Spronk HM 2010 Diesel engine exhaust initiates a sequence of pulmonary and cardiovascular effects in rats. J Toxicol 2010:206057; 10.1155/2010/206057 PMC2968117 21052503

[r30] Kraus U Schneider A Breitner S Hampel R Ruckerl R Pitz M 2013 Individual daytime noise exposure during routine activities and heart rate variability in adults: a repeated measures study. Environ Health Perspect 121 607 612; 10.1289/ehp.1205606 23512292 PMC3672128

[r31] Künzli N Jerrett M Garcia-Esteban R Basagaña X Beckermann B Gilliland F 2010 Ambient air pollution and the progression of atherosclerosis in adults. PLoS One 5 e9096; 10.1371/journal.pone.0009096 20161713 PMC2817007

[r32] Liao D, Creason J, Shy C, Williams R, Watts R, Zweidinger R (1999). Daily variation of particulate air pollution and poor cardiac autonomic control in the elderly.. Environ Health Perspect.

[r33] Magari SR, Hauser R, Schwartz J, Williams PL, Smith TJ, Christiani DC (2001). Association of heart rate variability with occupational and environmental exposure to particulate air pollution.. Circulation.

[r34] Malik M (1996). Heart rate variability: standards of measurement, physiological interpretation, and clinical use. Task Force of the European Society of Cardiology and the North American Society of Pacing and Electrophysiology.. Eur Heart J.

[r35] Meier R, Cascio WE, Danuser B, Riediker M (2013a). Exposure of highway maintenance workers to fine particulate matter and noise.. Ann Occup Hyg.

[r36] Meier R, Clark K, Riediker M (2013b). Comparative testing of a miniature diffusion size classifier to assess airborne ultrafine particles under field conditions.. Aerosol Sci Tech.

[r37] Miller MR, Hankinson J, Brusasco V, Burgos F, Casaburi R, Coates A (2005). Standardisation of spirometry.. Eur Respir J.

[r38] Peters A, Frohlich M, Doring A, Immervoll T, Wichmann HE, Hutchinson WL (2001). Particulate air pollution is associated with an acute phase response in men; results from the MONICA-Augsburg study.. Eur Heart J.

[r39] Peters A, von Klot S, Heier M, Trentinaglia I, Hormann A, Wichmann HE (2004). Exposure to traffic and the onset of myocardial infarction.. N Engl J Med.

[r40] Pieters N, Plusquin M, Cox B, Kicinski M, Vangronsveld J, Nawrot TS (2012). An epidemiological appraisal of the association between heart rate variability and particulate air pollution: a meta-analysis.. Heart.

[r41] Pope CA, Verrier RL, Lovett EG, Larson AC, Raizenne ME, Kanner RE (1999). Heart rate variability associated with particulate air pollution.. Am Heart J.

[r42] Ridker PM, Hennekens CH, Buring JE, Rifai N (2000). C-reactive protein and other markers of inflammation in the prediction of cardiovascular disease in women.. N Engl J Med.

[r43] Riediker M, Cascio WE, Griggs TR, Herbst MC, Bromberg PA, Neas L (2004). Particulate matter exposure in cars is associated with cardiovascular effects in healthy young men.. Am J Respir Crit Care Med.

[r44] Rioux CL Tucker KL Mwamburi M Gute DM Cohen SA Brugge D 2010 Residential traffic exposure, pulse pressure, and C-reactive protein: consistency and contrast among exposure characterization methods. Environ Health Perspect 118 803 811; 10.1289/ehp.0901182 20123638 PMC2898857

[r45] Samet JM, Rappold A, Graff D, Cascio WE, Berntsen JH, Huang YCT (2009). Concentrated ambient ultrafine particle exposure induces cardiac changes in young healthy volunteers.. Am J Respir Crit Care Med.

[r46] Schiller B, Hammer J, Barben J, Trachsel D (2009). Comparability of a hand-held nitric oxide analyser with online and offline chemiluminescence-based nitric oxide measurement.. Pediatr Allergy Immunol.

[r47] Schwartz J (1999). Air pollution and hospital admissions for heart disease in eight U.S. counties.. Epidemiology.

[r48] Schwartz J, Laden F, Zanobetti A (2002). The concentration-response relation between PM_2.5_ and daily deaths.. Environ Health Perspect.

[r49] Selander J, Nilsson ME, Bluhm G, Rosenlund M, Lindqvist M, Nise G (2009). Long-term exposure to road traffic noise and myocardial infarction.. Epidemiology.

[r50] Timonen KL, Vanninen E, de Hartog J, Ibald-Mulli A, Brunekreef B, Gold DR (2006). Effects of ultrafine and fine particulate and gaseous air pollution on cardiac autonomic control in subjects with coronary artery disease: the ULTRA study.. J Expo Sci Environ Epidemiol.

[r51] Tomei G, Fioravanti M, Cerratti D, Sancini A, Tomao E, Rosati MV (2010). Occupational exposure to noise and the cardiovascular system: a meta-analysis.. Sci Total Environ.

[r52] van Kempen E, Babisch W (2012). The quantitative relationship between road traffic noise and hypertension: a meta-analysis.. J Hypertens.

[r53] van Kempen EE, Kruize H, Boshuizen HC, Ameling CB, Staatsen BA, de Hollander AE (2002). The association between noise exposure and blood pressure and ischemic heart disease: a meta-analysis.. Environ Health Perspect.

[r54] Weichenthal S Kulka R Dubeau A Martin C Wang D Dales R 2011 Traffic-related air pollution and acute changes in heart rate variability and respiratory function in urban cyclists. Environ Health Perspect 119 1373 1378; 10.1289/ehp.1003321 21672679 PMC3230442

[r55] Wu S Deng F Niu J Huang Q Liu Y Guo X 2010 Association of heart rate variability in taxi drivers with marked changes in particulate air pollution in Beijing in 2008. Environ Health Perspect 118 87 91; 10.1289/ehp.0900818 20056565 PMC2831973

[r56] Yeatts K Svendsen E Creason J Alexis N Herbst M Scott J 2007 Coarse particulate matter PM_2.5–10_ affects heart rate variability, blood lipids, and circulating eosinophils in adults with asthma. Environ Health Perspect 115 709 714; 10.1289/ehp.9499 17520057 PMC1867980

